# Brain regions important for recovery after severe post-stroke upper limb paresis

**DOI:** 10.1136/jnnp-2016-315030

**Published:** 2017-06-22

**Authors:** Jane M Rondina, Chang-hyun Park, Nick S Ward

**Affiliations:** 1 Sobell Department of Motor Neuroscience and Movement Disorders, Institute of Neurology, University College London, London, UK; 2 Department of Neurology, Ewha Medical Research Institute, Ewha Womans University School of Medicine, Seoul, Republic of Korea; 3 Rehabilitation, The National Hospital for Neurology and Neurosurgery, London, UK; 4 UCL Partners Centre for Neurorehabilitation, London, UK

**Keywords:** motor recovery, stroke, proportional recovery, corticospinal tract, lesion likelihood, support vector machine.

## Abstract

**Background** The ability to predict outcome after stroke is clinically important for planning treatment and for stratification in restorative clinical trials. In relation to the upper limbs, the main predictor of outcome is initial severity, with patients who present with mild to moderate impairment regaining about 70% of their initial impairment by 3 months post-stroke. However, in those with severe presentations, this proportional recovery applies in only about half, with the other half experiencing poor recovery. The reasons for this failure to recover are not established although the extent of corticospinal tract damage is suggested to be a contributory factor. In this study, we investigated 30 patients with chronic stroke who had presented with severe upper limb impairment and asked whether it was possible to differentiate those with a subsequent good or poor recovery of the upper limb based solely on a T1-weighted structural brain scan.

**Methods** A support vector machine approach using voxel-wise lesion likelihood values was used to show that it was possible to classify patients as good or poor recoverers with variable accuracy depending on which brain regions were used to perform the classification.

**Results** While considering damage within a corticospinal tract mask resulted in 73% classification accuracy, using other (non-corticospinal tract) motor areas provided 87% accuracy, and combining both resulted in 90% accuracy.

**Conclusion** This proof of concept approach highlights the relative importance of different anatomical structures in supporting post-stroke upper limb motor recovery and points towards methodologies that might be used to stratify patients in future restorative clinical trials.

## Introduction

Stroke is one of the the most common causes of physical disability worldwide and about 80% of stroke survivors experience impairment of movement on one side of the body.^1^ Hand and arm impairment in particular is often persistent, disabling and a major contributor to reduced quality of life.^2^ The main predictor of long-term outcome of upper limb function is the level of initial impairment.^3^ This can be quantified as the proportional recovery rule which states that by 3 months, patients with stroke will recover about 70% of the initial upper limb motor impairment that has been observed on day 3 post-stroke.^4–6^ The prediction works extremely well for those presenting with mild to moderate upper limb impairment, but in only about half of those with initially severe upper limb impairment.^4–6^ In the other half, patients do worse than predicted, that is, there is a failure of proportional recovery. A key question then is, what is the difference between patients with stroke matched for initial severity who go on and have different recovery trajectories? The answer to this will point to the factors that are important for the dynamic process of recovery independent from the causes of initial impairment.

One possibility is the anatomy of the damage may be different in each group. A number of recent studies have proposed that the corticospinal tract (CST) plays a decisive role in this categorical difference^7–11^ as cortical reorganisation for improved motor function ultimately requires access for cortical motor areas to muscles. However, CST lesion load correlates with initial motor impairment,^12^ which is the major predictor of long-term outcome. It is therefore reasonable to ask how much CST lesion load can improve prediction of long-term outcome over and above initial severity. Furthermore, most of the patients involved in these studies had suffered from subcortical stroke and recent work has suggested that taking account of cortical damage after stroke can improve prediction of the motor clinical consequences.^13 14^

In this study, we investigated 30 patients with chronic stroke with a range of lesion locations (cortical and/or subcortical involvement) known to have presented with severe initial upper limb impairment but who had gone on to have quite different recovery trajectories. We applied a support vector machine approach to data representing lesion likelihood derived from structural T1-weighted MRI to answer the following questions. First, how accurately can patients with stroke with severe initial upper limb impairment be classified as having either good or poor recovery using only data extracted from whole brain structural MRI? Second, which brain regions contribute most to the classification? The results have the potential to transform how prediction of long-term upper limb outcome after stroke is achieved in routine clinical practice in future. The ability to easily and accurately predict outcome with standard clinical neuroimaging would have important implications for planning of treatment but also for stratification in future trials of restorative therapies.^15^

## Methods

### Experimental design

Patients with stroke provided full written consent to take part in this study in accordance with the Declaration of Helsinki. The study was approved by the Joint Ethics Committee of the Institute of Neurology, University College London (UCL) and National Hospital for Neurology and Neurosurgery, UCL Hospitals National Health Service (NHS) Foundation Trust, London. Patients were recruited from the Sobell Stroke Database. This database comprises first time adult stroke patients presenting with some level of motor impairment who have consented to be contacted about participating in research studies.

The database was screened for patients with severe initial upper limb impairment following first and only stroke (ischaemic or haemorrhagic) as assessed using the SAFE score,^10^ which used the Medical Research Council scale to grade shoulder abduction (SA) and finger extension (FE) on a scale of 0–5. Patients were eligible for this study if they had a SAFE score of 0 (no muscle activity) at 72 hours poststroke irrespective of whether reperfusion therapy was administered or not. The SAFE score was recorded in the medical notes (UCL Hospitals NHS Foundation Trust) within 72 hours of stroke onset in all cases.

In this study, we required two further pieces of information on each patient: (1) upper limb function in the chronic stage of stroke (at least 6 months since stroke); (2) T1-weighted structural whole brain MRI, which in these patients had been performed at the time of chronic upper limb function being scored. These data would allow us to retrospectively determine the extent of recovery (not just clinical outcome) in each individual.

Upper limb motor impairment in the chronic stage was assessed using four tests: Action Research Arm Test,^16^ grip strength,^17^ Motricity Index and Nine-Hole Peg Test.^18^ A single representative measure was calculated using principal component analysis (PCA) of the four motor scales to account for floor and ceiling effects in individual scores. PCA is mathematically defined as an orthogonal linear transformation that converts the data to a new coordinate system such that the greatest variance by some projection of the data comes to lie on the first coordinate (called the first principal component), the second greatest variance on the second coordinate and so on. Patients were ranked according to the first principle component of the PCA. The top 40% and the bottom 40% of patients were then included as good and poor recoverers, respectively. The middle 20% were excluded to ensure that we were investigating two groups of patients with clearly distinct recovery profiles, despite all presenting with the same level of initial severity.

Anatomical T1-weighted volumetric MRI high-resolution images were acquired using a 3T Allegra scanner (Siemens AG, Erlangen, Germany) with the following protocol: number of slices=176, slice thickness=1 mm, matrix size=224×256, in-plane resolution=1 mm×1 mm. The origin of each image was set at the anterior commissure. Images from patients that had injury predominantly in the left hemisphere were flipped in relation to the mid-sagittal plane so that all scans presented lesion in the right hemisphere.

### Data representation—obtaining lesion likelihood images

Images where each voxel contains a measure representing the probability of being part of injured tissue were derived from the T1-weighted volumetric MRI scans using an automatic method for detection of outlier voxels.^19^ This approach is based on the assumption that lesions are characterised as atypical voxels regarding expected brain tissues (grey matter, white matter and cerebrospinal fluid). The procedure uses the unified segmentation–normalisation approach^20^ modified to include an extra tissue to account for the perturbation introduced by lesions. In the resultant image, each voxel is assigned a value between 0 and 1 that represents its probability of being part of a lesion. We call this representation lesion likelihood.

It is important to note that the lesion likelihood is different from lesion load, another way of extracting data from structural images, which has been commonly used in studies that predict poststroke outcome.^21–25^ The lesion load is a summarised measure that represents the proportion of voxels in the brain (or within an anatomical structure) that are considered to be injured. Thus, it requires a procedure to delineate the lesion. Using lesion likelihood data is superior to lesion load data in predicting motor impairment after stroke, irrespective of the region of interest used.^14^

**Figure 1:**
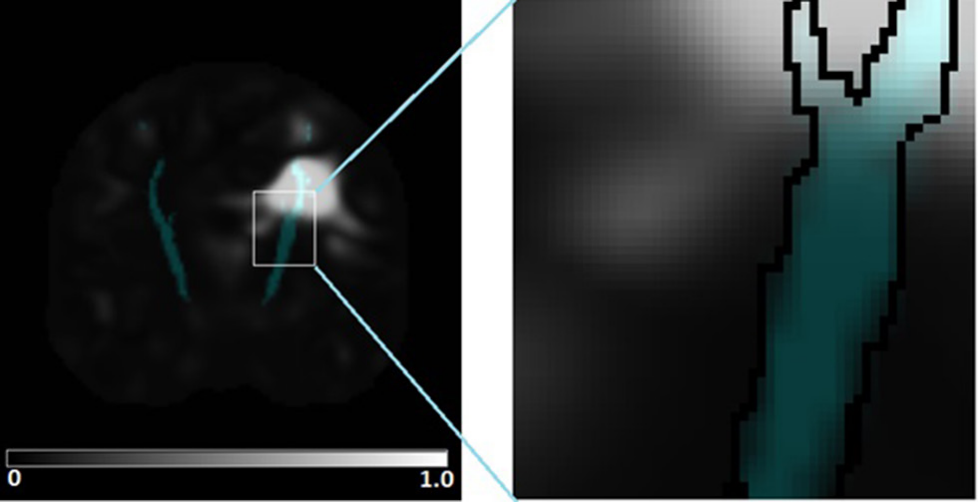
Data representation. In the figure to the left, a mask corresponding to the corticospinal tract is overlaid on an image obtained through lesion likelihood. Each voxel corresponds to a value between 0 and 1 encoding the probability of being part of injured tissue. The enlarged section of the image in the figure to the right shows that each voxel within a region of interest corresponds to a particular feature in the multivariate analysis.

Figure 1 displays an example of lesion likelihood image. The CST mask (represented in red) is circled in the enlarged image on the right side to show that each voxel inside the mask corresponds to a feature (as opposed to the lesion load, where a single value (or feature) would be extracted).

### Machine learning classification

One of the most common objectives of machine learning algorithms is classification, which attempts to assign each input value to one of a given set of classes. Classification analysis has become increasingly popular in clinical research, with potential to contribute to diagnosis, prognosis and prediction of treatment response. In brief, classification methods work as follows: given a set of training examples, each one known to belong to a specific category (class), the training algorithm learns a function based on the values of each variable (feature). The decision function learnt from the training set is used to classify a new example (ie, to predict the category to which it belongs) based on the values of its variables. In neuroimaging, features usually correspond to voxels derived from a brain scan or some form of summarisation of groups of voxels.

Using lesion likelihood data as input features, we classified patients as good recoverers (GR) or poor recoverers (PR) by applying linear Support Vector Machine (SVM)^26^ implemented in LIBSVM library^27^ used in Pattern Recognition for Neuroimaging Toolbox.^28^ The problem of obtaining a decision function in SVM consists in finding a hyperplane (a plane in a hyperspace), which has the largest margin between the closest examples across classes (called support vectors). When data from a new patient (not used to train the machine) is applied to the function, the class to which it belongs is determined.

**Figure 2:**
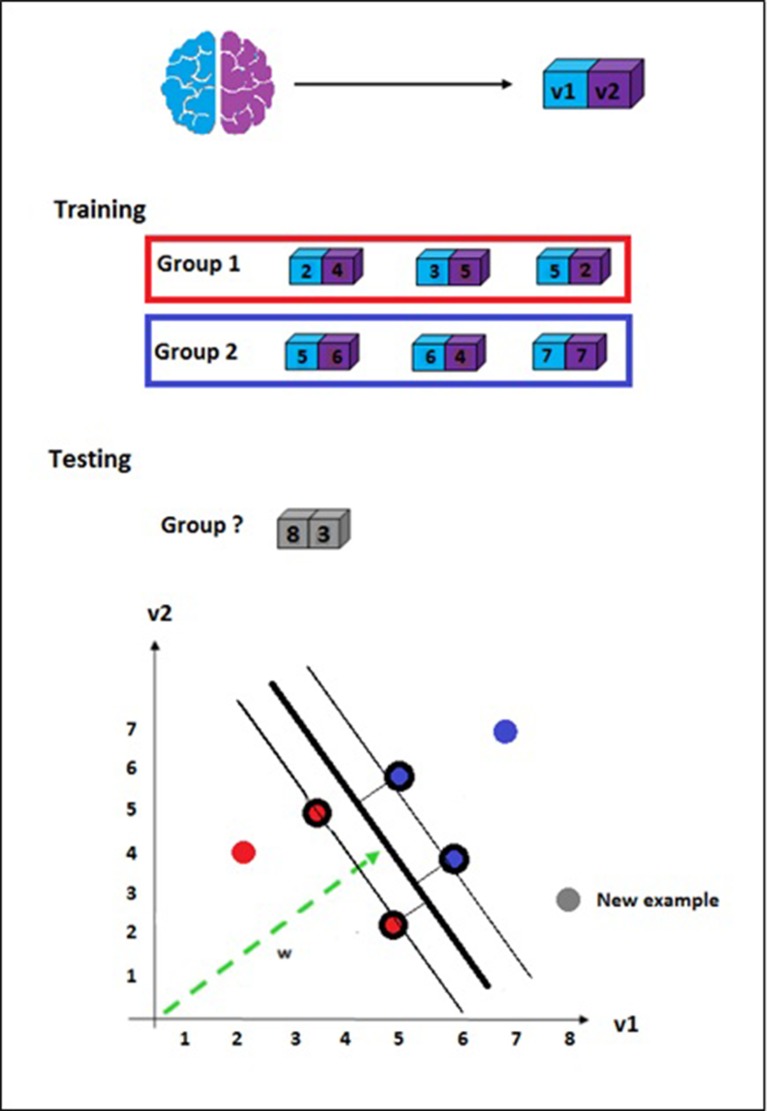
Support Vector Machine illustration. Simplified representation of a training set with two groups, each one comprised three examples. Each example has only two features representing voxels v1 and v2. The examples are projected in a space *R^n^*, where *n* is the number of features. Although the illustration represents a two-dimensional space, in a real high-dimensional problem with potentially thousands of features, the examples are projected in a hyperspace. The choice of the decision function among all hyperplanes that can separate the training set in classes is based on the maximisation of the margin between the closest examples (support vectors, circled in the figure). The decision regarding the class to which the new example belongs depends on the values of its features applied to the decision function.

Figure 2 presents a simplified representation of SVM to classify examples (patients) based on features (voxels from brain images). In the simplification, we represented each example with two features only (eg, one voxel corresponding to each hemisphere) to be able to illustrate it in a two-dimensional graph.

In our context, we have a binary classification (two classes, corresponding to GR and PR patients). The number of examples (n) is 30 and the number of features (p) corresponds to the number of voxels in the analysis. Given the training data (*x_i_, y_i_*) for *i=1…*n, with *y_i_* ϵ {*−1,1*}, a classifier *f(x)* is learnt such that:

$$f\left(x\right)=\left\{ \begin{array}{c} -1\text{, }x<0\\ 1\text{, }x\geq 0 \end{array}\right.$$

A linear classifier has the form: *f(x) = wT x + b*, where *w* is known as the weight vector and *b* is a bias. Considering only two features, the discriminant function *f(x)* would be a line, as illustrated in figure 2. In a real analysis, however, each feature vector may contain thousands of voxels, thus being represented in a hyperspace.

The weight vector is a linear combination of the support vectors. A weight is assigned to each voxel, with larger weights indicating voxels of higher relevance for obtaining the discriminant hyperplane. Considering that a positive and a negative label are associated to each group (ie, +1=  poor recoverers; −1= good recoverers), a positive weight assigned to a voxel means a higher relative level of lesion likelihood in that voxel for poor recoverers compared with good recoverers in the support vectors, and a negative weight means that lesion likelihood was higher for good recoverers. It should be noted that, as the weights are defined by the support vectors that are related to the placement of the discriminant hyperplane, both magnitude and the sign (positive or negative) of the weights are defined in a multivariate way and the discrimination is based on the complete pattern of voxels. Therefore, it is not appropriate to draw local inferences about particular voxels. Instead, weights of individual voxels should be interpreted within the context of their contribution to a wider discriminating pattern.

### Delimiting regions of interest

To investigate the involvement of the CST and other ROIs, we defined binary masks to restrict voxels anatomically. A mask corresponding to the CST was obtained by probabilistic tractography from nine age-matched healthy volunteers in a previous study.^29^ Another mask was defined selecting a subset of ROIs from the Automated Anatomical Labeling (AAL) atlas^30^ that correspond to regions expected to be related to motor and sensorial function according to literature.^31–34^ The regions are the following (bilaterally): postcentral gyrus, precentral gyrus, supplementary motor area, superior frontal gyrus, middle frontal gyrus, inferior and superior parietal regions, thalamus, caudate, putamen and pallidum. We also performed a classification with a mask combining both the CST and the subset of the AAL ROIs selected. It is important to note that there was an intersection of 1128 voxels between the CST and the AAL ROIs selected. These voxels were removed from the motor ROIs mask, so that the both masks are disjoint.

### Statistical analysis

To evaluate the generalisation ability of the model, the dataset was partitioned into training and testing sets using a ‘Leave–one-pair–out’ cross-validation approach, with one patient from each group left out for test at each iteration. The performance of the analysis was described through the percentage of true positives and true negatives (correctly classified PR and GR patients, respectively). Statistical significance was tested using permutation, a non-parametric approach through which the frequency distribution under the null hypothesis is obtained combining random rearrangements of the labels across the examples. As the correlation between examples and labels is destroyed, one expects the classification accuracy with permuted labels to be close to chance (around 50%). The number of permutations repeated in each analysis was 10 000 times.

## Results

Thirty-eight patients with stroke with a SAFE score of 0 at presentation were found (from 150 patients) in the Sobell Stroke Database. When these patients were ranked according to their current upper limb motor score, we excluded the middle 20% (8 patients) to ensure clearly distinct recovery trajectories in our two groups. The remaining 30 patients (mean age 55.4 (SD 10.05) years, 12 females) were included in the analysis. Table 1 presents the description of demographic and clinical characteristics of each group. The continuous measures (age, time since stroke and the motor scales) were described through mean and standard variation for each group and the statistical difference between groups was tested using the Wilcoxon rank-sum test for each of these variables. The gender was described through the number of males and females, and the statistical significance between groups was tested using χ^2^. The lesion prevalence map for all patients is shown in online supplementary figure 1.

10.1136/jnnp-2016-315030.supp1 Supplementary data

**Table 1:** Demographic and clinical characteristics of each group of patients: poor recoverers (PR) and (good recoverers (GR)

	PR n=15	GR n=15	*p* Value
Age: mean (SD)	59.1 (7.2)	51.7 (10.8)	0.04*
Gender, number of patients: M (F)	10 (5)	8 (7)	0.46**
Time since stroke: mean (SD) and range (months)	40.7 (42.6) 6–165	31.3 (28.2) 6–116	0.74*
Ratio of ischaemic to primary intracerebral haemorrhagic stroke	12:3	13:2	–
ARAT: mean (SD) (max 57)	32.9 (8.5)	52.7 (5.49)	<0.01*
Grip mean: (SD) (% unaffected side)	41.4 (14.3)	74.6 (18.57)	<0.01*
Motricity index: mean (SD) (% unaffected side)	65.2 (11.4)	91.9 (4.2)	<0.01*
NHPT: mean (SD) (% unaffected side)	5.8 (5.9)	53.1 (23.7)	<0.01*

*p Value for Wilcoxon rank-sum test.

**p Value for χ^2^ test.

ARAT, Action Research Arm Test; NHPT, Nine-Hole Peg Test.

In the first analysis, we used all voxels in the whole brain (without applying masks to restrict anatomical ROIs). With this approach, it was possible to correctly classify 73% of the poor recoverers (true positive) and 87% of the good recoverers (true negative). Thus, the classification accuracy (average between true positive and true negative) was 80% (p=0.0102, given by permutation).

Restricting the voxels using the CST mask, 67% of the poor recoverers and 80% of the good recoverers were correctly classified (accuracy 73%, p=0.0260). Using the motor ROIs mask, 87% of the poor recoverers and 87% of the good recoverers were correctly classified (accuracy 87%, p=0.0006). The best result was obtained combining both masks (CST and motor ROIs), with 87% of the poor recoverers and 93% of the good recoverers correctly classified (accuracy 90%, p=0.0002) (table 2).

**Table 2:** Classification results

Features delimitation	T(PR)	T(GR)	Acc	*p* Value
Whole brain	73%	87%	80%	0.0102
CST mask	67%	80%	73%	0.0260
Motor ROIs mask	87%	87%	87%	0.0006
CST + Motor ROIs mask	87%	93%	90%	0.0002

Acc, accuracy (average between T(PR) and T(GR)); CST, corticospinal tract; *p*, statistical significance of the results (given by 10 000 permutations of the labels); ROI, region of interest; T(PR), proportion of poor recoverers correctly classified; T(GR), proportion of good recoverers correctly classified.

**Figure 3:**
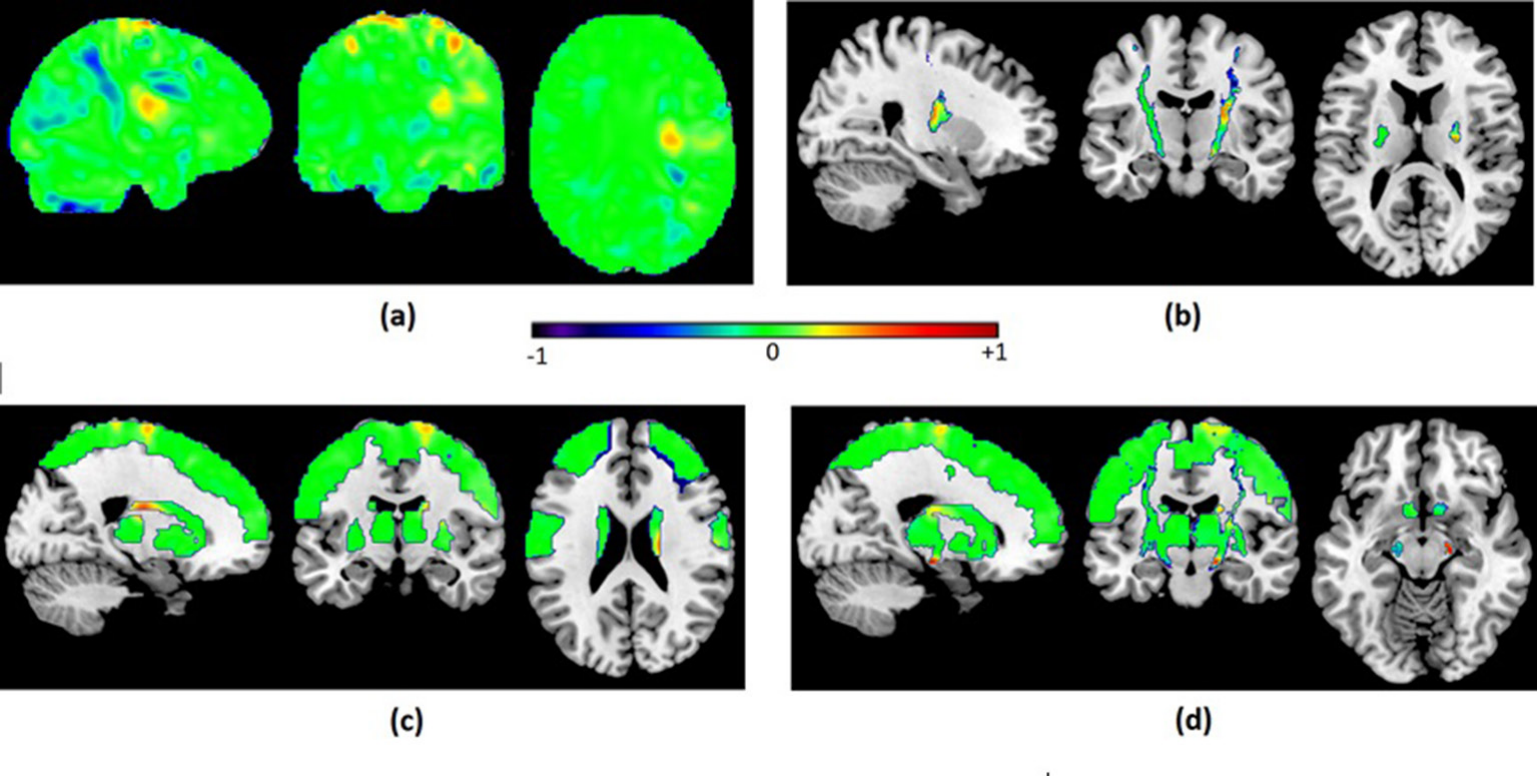
Discriminant maps resulting from classification of PR versus GR using: (A) the whole brain; (B) a CST mask; (C) motor ROIs mask; (D) CST + motor ROIs mask. The weight vector represents the relative relevance of each voxel to classify the groups. Positive values (represented in green towards red) mean a higher relative level of lesion likelihood for poor recoverers compared with good recoverers in the support vectors, and negative weights mean that lesion likelihood was higher for good recoverers. CST, corticospinal tract; GR, good recoverers; PR, poor recoverers.

Figure 3 shows the discriminant maps for all analyses. The maps were obtained using the SVM weight vector averaged across all cross-validation folds and represent the relative relevance of each feature (voxel) to classify the groups. Although it is not possible to make inferences regarding the relevance of specific locations based on the weight vectors due to the multivariate nature of the analysis, it is observable that there are some aggregations of voxels of similar weights (both positive and negative) and that the patterns of weights differ between left and right hemispheres in all analysis.

## Discussion

We have used a support vector machine approach to classify patients with stroke presenting with severe upper limb motor impairment as good or poor recoverers using only structural brain images containing voxel-wise information about the probability of damage. There are three key findings: (1) Classifying patients with stroke good or poor recoverers using only information from structural brain images is feasible; (2) Accurate classification was possible when using lesion likelihood information from just the CST, supporting the idea that a lower level of CST injury is important for recovery independent of its effect on initial severity of motor impairment; (3) However, classification using only voxels within regions commonly associated with motor and sensory function led to a substantial improvement in the classification accuracy in comparison with both the whole brain and the CST only models. This proof of concept approach highlights the relative importance of different anatomical structures in supporting upper limb motor recovery after stroke over and above their effect on initial impairment.

The proportional recovery rule principally demonstrates that the most important predictor of long-term upper limb outcome is initial severity. However, the presence of non-fitters to this rule indicates that other factors are important for understanding the dynamic process of recovery of motor function. We have not sought to replicate the proportional recovery result but have simply exploited the finding that patients presenting with severe impairment can have quite different recovery patterns (at the impairment level). This has allowed us to examine the anatomical factors important for upper limb motor recovery independent of their effects on initial severity. Previous hypotheses concerning why some patients fail to achieve good recovery have focused on the anatomy of the damage, in particular CST damage.^9 12 35^ Because CST damage correlates with initial upper limb severity and because many of the patients previously studied did not have cortical damage, we have extended these findings to include consideration of damage to cortical regions, and in particular, sensorimotor-related cortical regions. Our results highlight the relative importance of quantifying damage in these non-CST motor-related regions and argue for their inclusion to be tested in future predictive models for long-term upper limb outcome.

The proportional recovery rule has to date used the upper limb Fugl-Meyer scale. However, we did not have access to initial upper limb Fugl-Meyer scores, and so the results are not directly comparable to those previous studies.^4–6^ However, here we were not seeking to replicate the proportional recovery rule, but rather investigate why some patients who present with severe upper limb impairment recover and why some fail to recover, given that this difference could not be explained by initial severity. The patients in this study all had severe upper limb impairment according to a SAFE score of 0.^10^ It should be noted that the SAFE score has been obtained retrospectively. However, rather than relying on patient recall, the MRC grading scale score for SA and FE was recorded in the medical notes at the time of assessment in all cases and so is likely to be accurate. In relation to the outcomes in the chronic stage, inspection of the mean scores demonstrates our key requirement, namely that patients in each group have very different recovery trajectories. Despite not using the Fugl-Meyer score, our cohort represents a group of patients with stroke with severe upper limb impairment at 72 hours poststroke, who then separate into those with good recovery and those with poor recovery. As such, our results are still relevant for examining the residual structural brain architecture that supports upper limb recovery.

An important advantage of using patterns of voxels representing lesion likelihood instead of quantifying the lesion load is that the patterns take into account how the lesion spreads throughout anatomical regions, while lesion load presents a single value representing the proportion of damage in an anatomical structure.^14^ The same lesion load distributed according to different patterns can lead to different outcomes, especially in structures such as the CST that is particularly directional due to the tracts of fibres. Another advantage of using data represented as lesion likelihood is that there is no need to actually segment the lesions, avoiding a potential bias caused by threshold to obtain binary images of lesions.

Our findings support previous studies based on other methods that propose the important role of the CST as a biomarker for predicting recovery. Using less than 1% of the voxels of the whole brain, it was possible to classify good and poor recoverers with accuracy of 73%. However, the inclusion of other brain regions believed to be involved in reorganisation of motor functions led to an increase of the classification accuracy to 90%, suggesting that the information regarding the integrity of other cortical and subcortical areas potentially involved in sensorimotor function can also be important to predict recovery in patients who are severely impaired. It is also noticeable that the classification using the motor ROIs (excluding any intersection with the CST mask) resulted in better accuracy than using the CST itself.

There was a significant difference in age between our groups. The mean age of the poor recovery group was 7.4 years older than the good recovery group, although overall the average age was still below 60 and so this represents a relatively young stroke cohort. Increasing age confers a small risk of worse upper limb outcome overall,^3^ but age has not been reported to be a factor important for proportional recovery.^4–6^ Furthermore, recent evidence demonstrates that old patients benefit from high-intensity rehabilitation following stroke to the same degree as younger patients.^36^ It therefore remains to be seen whether this result holds true in a larger prospective cohort.

Our findings support the principle that accurate prediction of upper limb outcome using clinically acquired brain imaging data is a feasible and achievable goal in future. Our analysis relies only on automatic procedures and on structural T1-weighted MRI, an imaging protocol that is commonly acquired in clinical routine after stroke. This study is retrospective and requires further investigation in prospective studies with imaging data collected early after stroke. We will test whether the successful results obtained in the classification of different recovery trajectories of patients presenting with severe upper limb impairment (SAFE score=0) can be replicated in a longitudinal study, so that predictions performed early after stroke can contribute to more effective triage and stratification for neurorehabilitation. Beyond this however, the development of accurate models to predict functional outcomes after stroke is an important clinical priority. This work, together with work in different domains such as language,^37 38^ indicates that simple structural brain imaging together with automated analysis procedures can play an important role. Accurate predictive models will be important for planning of treatment but also for stratification in future trials of restorative therapies.
