# Supplementary figures and images for: Brain regions important for recovery after severe post-stroke upper limb paresis
Source: J Neurol Neurosurg Psychiatry. 2017 Jun 22;88(9):737–43. doi: 10.1136/jnnp-2016-315030 (PMC5561379; doi:10.1136/jnnp-2016-315030)

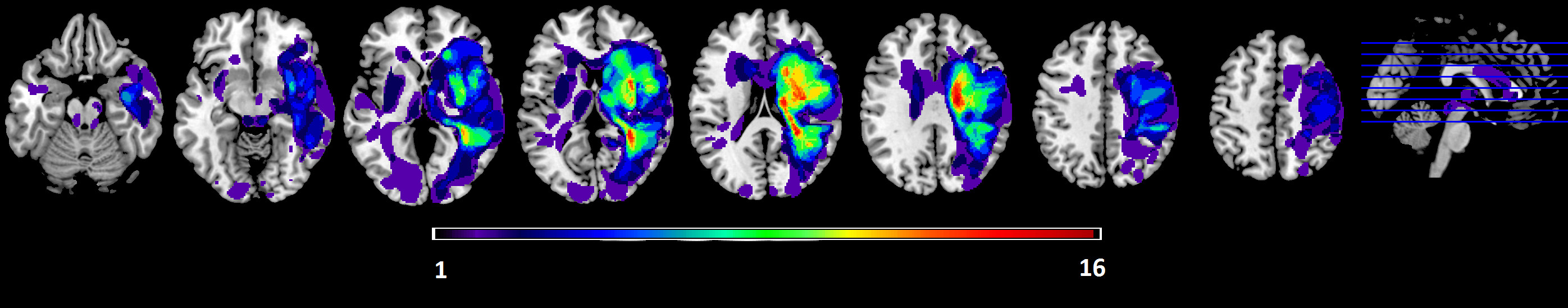

Supplement: Supplementary data [file jnnp-2016-315030supp001.jpg]
